# Current State and Impact of Behavioural Support Transition Units in Ontario Long‐Term Care Homes

**DOI:** 10.1002/alz70858_097430

**Published:** 2025-12-24

**Authors:** Jillian K McConnell, Katelynn Aelick

**Affiliations:** ^1^ brainXchange, North Bay Regional Health Centre, North Bay, ON, Canada; ^2^ Behavioural Supports Ontario Provincial Coordinating Office, North Bay, ON, Canada

## Abstract

**Background:**

Behavioural Support Transition Units (BSTUs) offer time‐limited assistance to individuals with dementia and older adults with other complex mental health conditions whose behavioural care needs surpass the capabilities of their current environment. Long‐term care home‐based BSTUs aim to reduce the occurrence and prevalence of residents' responsive behaviours, facilitating their transition to a lower level of care upon meeting clinical goals. In Ontario, 21 BSTUs currently operate with a combined capacity of 395 residents.

**Method:**

This presentation will synthesize the findings from the 2023 Ontario BSTU Environmental Scan alongside preliminary insights gleaned from the inaugural province‐wide dataset, encompassing data from Ontario's BSTUs. Data for both of these initiatives was collected directly from all BSTUs via electronic surveys and data collection forms.

**Results:**

The BSTU Environmental Scan provides a comprehensive analysis of the current landscape of Ontario's BSTUs, including resident demographics, environmental design, staffing models, consultation supports, and staff education. It identifies key areas for quality improvement, providing actionable insights to inform long‐term care professionals regarding patient referrals. The dataset highlights critical factors that influence the efficacy of BSTUs and uncovers trends relevant to improving care delivery.

**Conclusion:**

The dissemination of this information will contribute to a deeper collective understanding of the BSTU landscape, offering valuable insights for professionals in long‐term care who currently facilitate patient transitions to and from these units. Moreover, it will enhance awareness and inform healthcare professionals seeking to explore the role and impact of BSTUs on those living with cognitive impairment who are experiencing significant behavioural care needs, as well as the broader systems that support them.

